# Multi-Scale Entrainment of Coupled Neuronal Oscillations in Primary Auditory Cortex

**DOI:** 10.3389/fnhum.2015.00655

**Published:** 2015-12-09

**Authors:** M. N. O’Connell, A. Barczak, D. Ross, T. McGinnis, C. E. Schroeder, P. Lakatos

**Affiliations:** ^1^Cognitive Neuroscience and Schizophrenia Program, Nathan Kline InstituteOrangeburg, NY, USA; ^2^Department of Psychiatry, Columbia College of Physicians and SurgeonsNew York, NY, USA; ^3^Department of Psychiatry, NYU School of MedicineNew York, NY, USA

**Keywords:** entrainment, macaca mulatta, intracortical, neuronal oscillations, primary auditory cortex, tonotopic map

## Abstract

Earlier studies demonstrate that when the frequency of rhythmic tone sequences or streams is task relevant, ongoing excitability fluctuations (oscillations) of neuronal ensembles in primary auditory cortex (A1) entrain to stimulation in a frequency dependent way that sharpens frequency tuning. The phase distribution across A1 neuronal ensembles at time points when attended stimuli are predicted to occur reflects the focus of attention along the spectral attribute of auditory stimuli. This study examined how neuronal activity is modulated if only the temporal features of rhythmic stimulus streams are relevant. We presented macaques with auditory clicks arranged in 33 Hz (gamma timescale) quintets, repeated at a 1.6 Hz (delta timescale) rate. Such multi-scale, hierarchically organized temporal structure is characteristic of vocalizations and other natural stimuli. Monkeys were required to detect and respond to deviations in the temporal pattern of gamma quintets. As expected, engagement in the auditory task resulted in the multi-scale entrainment of delta- and gamma-band neuronal oscillations across all of A1. Surprisingly, however, the phase-alignment, and thus, the physiological impact of entrainment differed across the tonotopic map in A1. In the region of 11–16 kHz representation, entrainment most often aligned high excitability oscillatory phases with task-relevant events in the input stream and thus resulted in response enhancement. In the remainder of the A1 sites, entrainment generally resulted in response suppression. Our data indicate that the suppressive effects were due to low excitability phase delta oscillatory entrainment and the phase amplitude coupling of delta and gamma oscillations. Regardless of the phase or frequency, entrainment appeared stronger in left A1, indicative of the hemispheric lateralization of auditory function.

## Introduction

The most fundamental organizing principle of the auditory system at lower hierarchical levels of acoustic information processing is a faithful spatial representation of the auditory receptor surface in the cochlea (Schreiner and Winer, 2007). One of the likely reasons for the topographical organization of auditory information (tonotopy) across several earlier processing stages is that just like in signal processing (e.g., EEG analysis, photo or music editing), information can be best manipulated (e.g., filtered or sharpened) at high resolutions to enhance desired features. Once the information is compressed, at higher processing stages, only cruder aspects can be manipulated. Since frequency representation is condensed to a large degree already at the level of the second cortical processing stage in belt auditory cortex (Recanzone et al., 2000), theoretically, any refinement in the frequency composition of the auditory environment should ideally take place before this stage, in primary auditory cortex (A1) and subcortical structures.

Several studies have found that along with amplifying responses to task-relevant stimulus frequencies, attention also suppresses responses in neuronal ensembles tuned to the “ignored region” of the frequency spectrum (Fritz et al., 2003, 2005, 2007; Da Costa et al., 2013; Lakatos et al., 2013). This essentially represents a spectral filter mechanism that sharpens the frequency tuning of A1 by modulating auditory information across topographically organized neuronal ensembles. Two recent studies have found that when band limited attended auditory stimuli (pure tones) are presented rhythmically, a temporal filter component is superimposed on this spectral filter, in that frequency tuning will only be sharpened and attended frequency content will only be amplified at specific times, when relevant stimuli are predicted to occur (Lakatos et al., 2013; O’Connell et al., 2014). The mechanism of this spectrotemporal filter is the entrainment of ongoing neuronal oscillatory activity that represents spontaneous excitability fluctuations of the local neuronal ensemble. Oscillatory entrainment by the attended stimuli results in a predictive excitability modulation across all of A1. A key to the mechanism of the spectral filter component is that neuronal oscillations are entrained in counterphase across A1 neuronal ensembles tuned to relevant vs. irrelevant frequency content: while the excitability of neuronal ensembles tuned to attended frequencies is up-regulated preceding the predicted occurrence of stimuli to amplify responses, the excitability of neuronal ensembles around this region across most of A1 is down-regulated, as a means to suppress irrelevant inputs that temporally coincide with attended stimuli.

Contrasting with this counterphase entrainment in A1, an earlier study investigating the processing of rhythmic visual stimuli found that in the neuronal ensembles of the primary visual cortex (V1), ongoing oscillations were always entrained to their high excitability phases by attended stimuli (Lakatos et al., 2008). A likely reason for the difference between entrainment effects across topographically organized neuronal ensembles in A1 and V1 is that while the auditory studies used pure tones which excite only a subset of the tonotopically organized neuronal ensembles in A1, the visual study used flashes that were not confined in space, and thus activated a large proportion of the retinotopically organized V1 neuronal ensembles. Also, while in the auditory tasks the topographically mapped feature, the frequency of the stimuli, was task relevant, in the visual task, the topographically mapped feature, the spatial location of the flash was task irrelevant.

Based on these earlier results, the main hypothesis tested here was that if subjects attend to broadband auditory stimuli, whose frequency content is task irrelevant, ongoing oscillations in most auditory neuronal ensembles will be entrained to their high excitability, depolarizing phases, in order to predictively amplify incoming inputs. We also hypothesized that as a consequence, the overall effect of attention to these stimuli will be a response enhancement across most A1 sites independent of tonotopy. To test this, we presented auditory click-trains, since clicks have a broad frequency spectrum. Five clicks were used to create a click train (the standard stimuli), the same five clicks formed rarely occurring deviant, or target stimuli, and the only difference between the standard and deviant stimuli was in their temporal structure, rendering the frequency content task irrelevant. Contrary to our hypothesis, effects of attention in the auditory task differentiated across the tonotopic gradient in A1. Only neuronal ensembles tuned to around 11–16 kHz had a strong tendency to entrain to their high excitability phases on multiple timescales. The more common effect, observed over the remainder of A1 sites, was response attenuation, due to the predictive suppression of neuronal excitability on at least one of the task structure related timescales (intra and inter click-train) in most A1 sites. Additionally, we found evidence that entrainment was left lateralized, indicating that similar to humans (for a review, see Giraud and Poeppel, 2012), auditory cortical function might be lateralized even at the level of primary auditory cortex in non-human primates.

## Materials and Methods

### Subjects

In the present study, we analyzed the electrophysiological data recorded during 48 total penetrations of area A1 of the auditory cortex from two female macaques (*Macaca mulatta*; 22 penetrations from macaque A and 26 from macaque K) weighing 4–7 kg, who had been prepared surgically for chronic awake electrophysiological recordings. Prior to surgery, each animal was adapted to a custom fitted primate chair and to the recording chamber. All procedures were approved in advance by the Animal Care and Use Committee of the Nathan Kline Institute.

### Surgery

Preparation of subjects for chronic awake intracortical recording was performed using aseptic techniques, under general anesthesia, as described previously in Schroeder et al. (1998). The tissue overlying the calvarium was resected and appropriate portions of the cranium were removed. The neocortex and overlying dura were left intact. To provide access to the brain and to promote an orderly pattern of sampling across the surface of the auditory areas, cilux recording chambers (Crist Instruments) were positioned normal to the cortical surface of the superior temporal plane for orthogonal penetration of area A1, as determined by a pre-implant MRI. Together with socketed Plexiglas bars (to permit painless head restraint), they were secured to the skull with orthopedic screws and embedded in bone cement. A recovery time of minimum 6 weeks was allowed before the animal was head restrained and we began data collection.

### Electrophysiology

**Figure 1:**
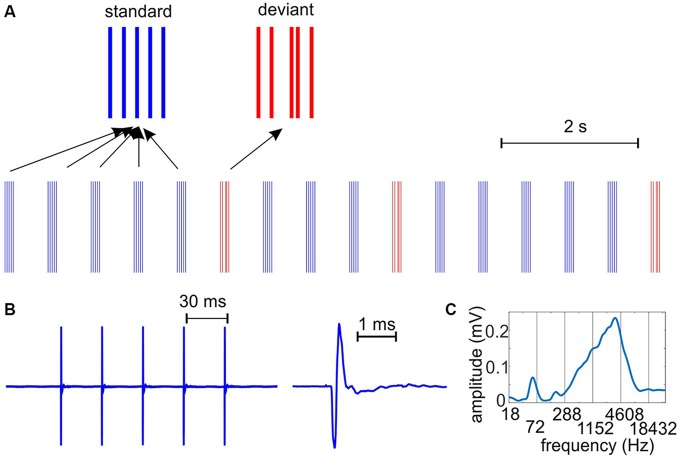
**The auditory stimulus stream. (A)** We presented auditory clicks with a hierarchically organized rhythmic structure on two time-scales: trains of five clicks had a repetition rate of 33 Hz, while the click-trains occurred at a rate of 1.6 Hz. Deviant (target in the engaged condition) stimuli had a different temporal structure: the third click was shifted in time. Standard and deviant click trains presented were the same in passive and engaged conditions. **(B)** To the left, a standard click-train recorded with a microphone and digitized at 100 kHz. To the right, the waveform of an individual “click” as emitted by the speakers. The duration of the bipolar pulse is approximately 0.3 ms. **(C)** Spectrogram of the click train shows a prominent peak around 3.5 kHz, corresponding to the 0.3 ms “wavelength”, and smaller peaks at harmonics of the repetition rate.

**Figure 2:**
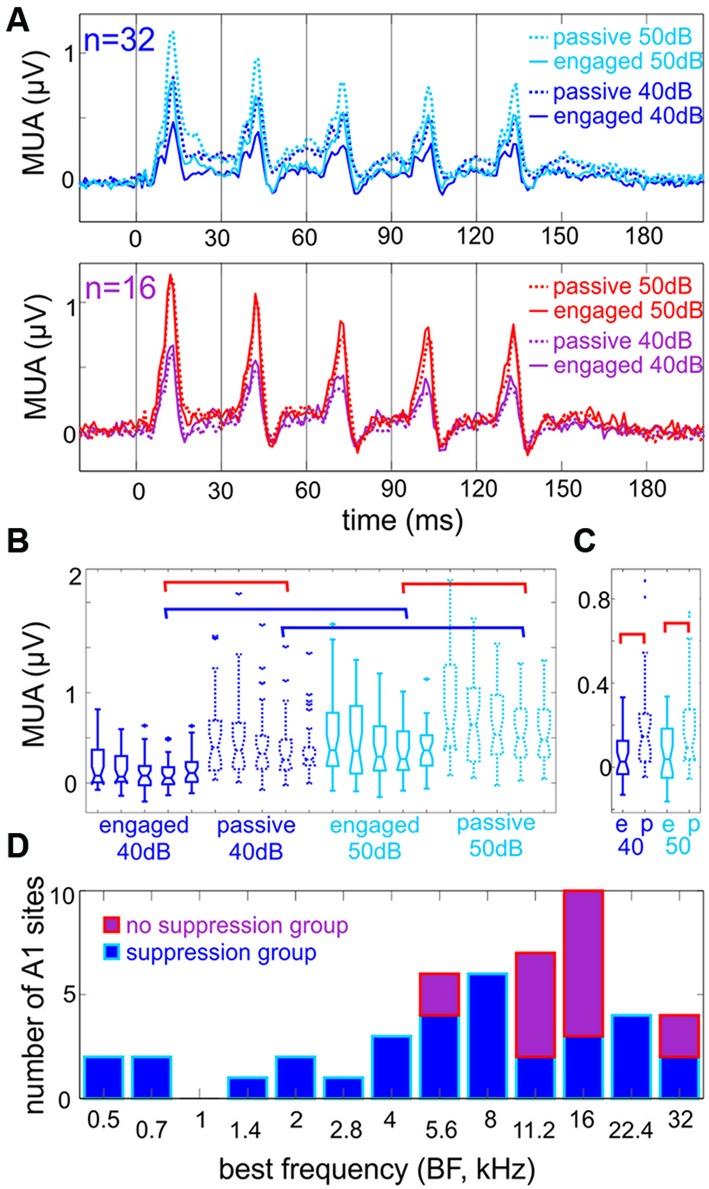
**Engagement generally suppresses responses to click-trains. (A)** Multiunit responses to click-trains. Top traces show averaged responses to click-trains presented at 40 and 50 dB loudness (dark vs. light blue), in engaged and passive trial blocks (solid vs. dotted traces), recorded in Auditory cortex (A1) sites where engagement resulted in a significant suppression of the Multiunit activity (MUA) response in the 6–160 ms time range. The bottom traces are pooled responses from A1 sites where engagement resulted in either no amplitude change (12 out of 16) or a significant enhancement of MUA in the same time range. **(B)** Boxplots show the amplitude of MUA responses of the suppressive group to the five clicks (8–14 ms after the onset of each click) presented at 40 and 50 dB loudness in engaged and passive trial blocks. The blue brackets indicate significant differences due to change in loudness, while red brackets indicate significant differences due to engagement in responses to the fourth click (Tukey’s test, *p* < 0.01). For clarity, not all significant differences are indicated by brackets. **(C)** Boxplots show the amplitude of “baseline” MUA (−10 to 5 ms around the onset clicks No 2–5) in different trial blocks (same as in **A** and **B**). Only engagement had a significant effect on baseline MUA indicated by the red brackets (Tukey’s test, *p* < 0.01), change in loudness did not. **(D)** The best frequency (BF) of sites with significant engagement related suppression (blue) and no significant suppression (violet). Note that the latter group of sites is concentrated in the high frequency tuned region of A1.

**Figure 3:**
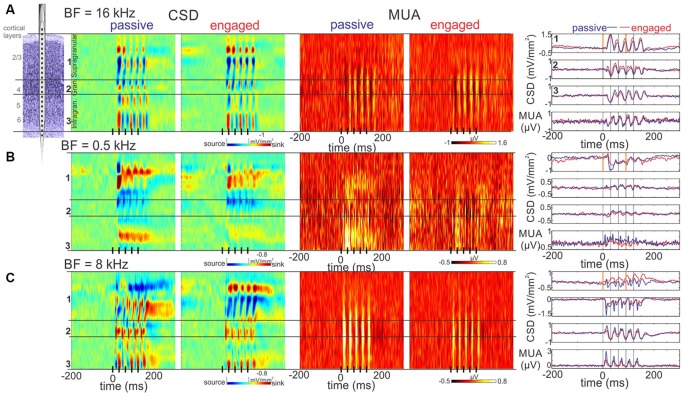
**Representative laminar response profiles. (A)** Color-maps to the left display laminar Current source density (CSD) profiles in response to click-trains from an A1 site with a BF of 16 kHz (no suppression group). The boundaries of the supragranular, granular and infragranular layers are marked by black horizontal lines. The click-train is represented by black lines on the *x*-axis. Note that there appear to be sink (red)-source (blue) pairs representing active and associated passive currents in each of the layers. Color-maps to the right display the concomitant laminar MUA responses, which show a somewhat larger amplitude response in the engaged condition. The upper three traces to the right of the color-maps show CSD responses from the supragranular, granular and infragranular electrode locations marked by numbers to the left of the CSD maps (for the selection of these see “Materials and Methods” Section). The apparently largest response amplitude difference in passive vs. engaged condition is at the supragranular electrode location. Note that at stimulus onset, the baseline is negative-trending (especially in the engaged condition), and at the onset of the fourth click (orange line at 90.9 ms) the gamma frequency waveform (SSR) in between is also negative-trending. The bottom traces display the MUA averaged across all layers recorded in passive vs. engaged conditions. **(B)** Same as **(A)**, but from a low BF A1 site with significant engagement related suppression (suppression group). As opposed to **(A)**, the supragranular CSD in the engaged condition is positive trending at 0 ms, indicating an opposite phase low frequency excitability modulation. The slope of the SSR at 90.9 ms is negative trending. **(C)** Same as **(A)**, but from a relatively high BF A1 site with significant engagement related suppression. While similar to **(A)**, the baseline is negative trending, the SSR waveform is positive trending at the onset of the fourth click. This latter effect appears much stronger in the engaged condition. Note that in all three sites, MUA at stimulus onset is oppositely trending to the supragranular CSD, indicating that similar to what previous studies found, a negative CSD trend signals increasing, while a positive CSD trend signals decreasing excitability.

Animals sat in a primate chair in a dark, isolated, electrically shielded, sound-attenuated chamber with head fixed in position, and were monitored with infrared cameras. Laminar profiles of neuroelectric activity were obtained simultaneously from left and right hemisphere auditory cortices using two linear array multi-contact electrodes (23 contacts, 100 μm intercontact spacing). Multielectrodes were inserted acutely through guide tube grid inserts, lowered through the dura into the brain, and positioned such that the electrode channels would span all layers of the cortex (Figure 1,2,3), which was determined by inspecting the laminar response profile to binaural broadband noise bursts. Neuroelectric signals were impedance matched with a pre-amplifier (10× gain, bandpass dc-10 kHz) situated on the electrode, and after further amplification (500×) they were recorded continuously in a 0.01–8000 Hz passband digitized at a sampling rate of 20 kHz and precision of 16-bits using custom made software in Labview. The signal was split into the field potential (0.1–300 Hz) and multiunit activity (MUA; 300–5000 Hz) range by zero phase shift digital filtering. MUA data was also rectified in order to improve the estimation of firing of the local neuronal ensemble (Legatt et al., 1980). One-dimensional current source density (CSD) profiles were calculated from the local field potential profiles using a three-point formula for the calculation of the second spatial derivative of voltage (Freeman and Nicholson, 1975). The advantage of CSD analysis is that CSD signals are not affected by volume conduction like the local field potentials, and they also provide a more direct index of the location, direction, and density of the net transmembrane current flow (Mitzdorf, 1985; Schroeder et al., 1998). At the beginning of each experimental session, after refining the electrode position in the neocortex, we established the best frequency (BF) of the recording site using a “suprathreshold” method (Steinschneider et al., 1995; Lakatos et al., 2005a). The method entails presentation of a stimulus train consisting of 100 random order occurrences of a broadband noise burst and pure tone stimuli presented at 50 dB loudness with frequencies ranging from 353.5 Hz–32 kHz in half octave steps (duration: 100 ms, rise/fall time: 5 ms, stimulus onset *asynchrony* (SOA) = 624.5 ms). Auditory stimuli for tonotopy and for the behavioral task were generated at 100 kHz sampling rate in Labview using a multifunction data acquisition device (National Instruments DAQ USB-6259), and presented through SA1 stereo amplifiers coupled to FF1 free field speakers (Tucker-Davis Technologies). Loudness was calibrated using measurements made with an ACO Pacific PS9200/4012 calibrated microphone system.

### Behavioral Task and Stimuli

Using an auditory task with broadband stimuli, the goal of the present set of experiments was to examine the effect of engagement on the entrainment of neuronal oscillations on multiple time-scales and on auditory responses. We presented the subjects rhythmic streams of click-trains (Figure 1A): the click-trains consisted of five clicks (40 or 50 dB SPL loudness), generated by driving the speakers with five 0.1 ms square waves that were arranged 30.3 ms apart. The click-trains were repeated every 624.5 ms (constant SOA). In this rhythmic stream of standard, frequently presented click-trains, deviant click-trains occurred at 2–6 s random time intervals. Deviant click-trains only differed in their temporal structure: the third click was delayed by 15–30.3 ms depending on the subject’s performance which we tried to keep between 60–80% correct. To engage the monkeys in detecting deviant or target click-trains, in the beginning of training, 0.25–1 ml juice reward was delivered to them simultaneously with each deviant through a tube. The tube was positioned such that the monkeys had to stick out their tongue in order to get the juice. Licking was monitored using a simple contact detector circuit (Slotnick, 2009), the output of which was continuously recorded together with the timing of standard and deviant tones for offline analyses via a multifunction data acquisition device (National Instruments DAQ USB-6259) in Labview. In this phase of training the third click in the deviant click-trains was shifted by 30.3 ms corresponding to a missing third click. After two sessions, the juice reward was omitted on every 10th deviant. If the monkeys licked on these deviants without a paired juice reward, signaling that they were engaged in the auditory task, we omitted the reward on 20% of the deviants, and also gradually decreased the shift of the third click when the monkey’s performance increased to around 80%. For one of the subject’s the shift was decreased to 15 ms in the last experiments, while for the other monkey the shift was never below 25 ms. We only analyzed data related to standard stimuli that preceded deviants on which the subjects licked (whether or not the deviants were paired with juice). Further, we only analyzed CSD and MUA data related to standards that followed the deviant by a minimum of four stimulus positions, to avoid artifacts related to licking and to ensure that subjects re-engaged in the task (deviants could not occur for 2 s following a deviant/target).

Besides the engaged, auditory task condition, we recorded data during the presentation of the same stimuli in a passive condition, when the juicer was removed, and the subjects had no auditory or other task, but were quietly sitting in the recording chamber. Following the passive condition, we also recorded 3–5 min of spontaneous neuroelectric activity in the absence of stimuli presented.

### Data Analysis

Data were analyzed offline using native and custom-written functions in Matlab (Mathworks, Natick, MA, USA). After selective averaging of the CSD and MUA responses to the tones presented in the suprathreshold tonotopy paradigm, recording sites were functionally defined as belonging to AI or belt auditory cortices based on the sharpness of frequency tuning, the inspection of the tonotopic progression across adjacent sites, and relative sensitivity to pure tones vs. broad-band noise of equivalent intensity (Merzenich and Brugge, 1973; Rauschecker et al., 1997; Lakatos et al., 2005a). In the present study only recordings obtained from area A1 were analyzed. At the end of each animal’s experimental participation, functional assignment of the recording sites was confirmed histologically (Schroeder et al., 2001).

Utilizing the BF-tone related laminar CSD profile, the functional identification of the supragranular, granular and infragranular cortical layers in area A1 (Figure 3) is straightforward based on our earlier studies (Schroeder et al., 1998, 2001; Lakatos et al., 2005a, 2007). In the present study, we focused the analyses of ongoing and event related neuronal activity on the supragranular CSD with largest BF tone related activation (sink), and the MUA averaged across all layers. The reason for this selection is that both ongoing and entrained oscillatory activity are most prominent in the supragranular layer (Lakatos et al., 2005b, 2007, 2008), and they appear to reflect synchronous excitability fluctuations of the local neuronal ensembles across all layers, as evidenced by synchronous MUA amplitude fluctuation across the layers (O’Connell et al., 2011). Also, dominant delta frequency neuronal activity in all cortical layers is largely coherent with supragranular delta oscillatory activity, with varying but stable phase differences across cortical depths (Lakatos et al., 2005b, 2013; O’Connell et al., 2011, 2014).

To determine MUA response onset latencies, the MUA averaged across all cortical layers was used, and response onset was defined as the earliest significant [>2 standard deviation (SD) units] deviation of the averaged waveforms from their baseline (−50–0 ms), that was maintained for at least 5 ms.

For the analysis of ongoing and event related delta and gamma oscillatory activity, instantaneous power and phase in single trials were extracted by wavelet decomposition (Morlet wavelet) with 345 logarithmically spaced frequency steps ranging from 0.5–55 Hz. Oscillatory amplitudes were measured in spontaneous recordings and also in data recorded during stimulus presentation. In both cases, a continuous wavelet transform was performed on the entire recording, but in the latter case, only time-points during and following the presentation of standard tones (see above) were averaged. To characterize delta and gamma phase distributions related to stimulus presentations (trials), the wavelet transformed data were normalized (unit vectors), the data at corresponding time-points relative to each stimulus onset were averaged, and the length (modulus) of the resulting vector was computed (e.g., Lakatos et al., 2007). The value of the mean resultant length, also called inter-trial coherence (ITC) ranges from 0–1; higher values indicate that the observations (oscillatory phase at a given time-point across trials) are clustered more closely around the mean (i.e., phase distribution is biased) than lower values. Phase distributions were evaluated statistically using circular statistical methods. Significant deviation from uniform (random) phase distribution was tested with Rayleigh’s uniformity test. Pooled phase distributions were compared by a nonparametric test for the equality of circular means (Fisher, 1993; Rizzuto et al., 2006).

Independent of their waveform shape (frequency composition in the frequency domain), cyclically occurring events like the suprathreshold, “evoked type” response waveforms can artificially bias phase measures at the frequency that corresponds to the stimulus presentation rate (Lakatos et al., 2013; Zoefel and Heil, 2013). Since in some cases, visual inspection revealed a clear “evoked type” transient waveform in the supragranular CSD in response to the click-train (Figure 3, right traces), similar to our earlier studies in the case of responses to pure tones, we applied a linear interpolation to the single trials in the time interval of the evoked-type auditory response (5–150 ms), and determined delta phases in the interpolated data at click-train onset. For the same reason, we determined gamma phases at the time of the fourth click (90.9 ms) rather than at click-train onset, plus gamma entrainment most likely only develops after the third click.

## Results

We analyzed neuroelectric data recorded in 48 total A1 sites from two macaque monkeys (see Figure 2D for BF distribution). Ongoing and event related neuronal activity was recorded with linear array multielectrodes which spanned all cortical layers at each A1 recording site. To be able to directly compare simultaneous activity of left and right hemisphere A1 neuronal ensembles, the majority of the data (42 sites in 21 experiments) was obtained via simultaneous left and right A1 recordings targeting regions tuned to similar frequencies. To minimize the effects of volume conduction and more precisely define local laminar transmembrane current flow profiles (Freeman and Nicholson, 1975; Mitzdorf, 1985; Schroeder et al., 1998), we calculated one dimensional CSD from the field potentials and carried out most of our analyses on the CSD waveforms and concomitant MUA.

Auditory stimulus-related neuronal activity was recorded in two conditions in separate trial blocks: either the monkeys were attending to frequently repeating standard stimuli in order to detect deviants that differed from standards in their temporal structure (engaged), or they were passively listening to the same stimuli (passive). The SOA was a constant 624.5 ms in both conditions, corresponding to the average wavelength of dominant delta frequency oscillations in the ongoing neuronal activity of primary auditory cortex (Lakatos et al., 2005b). Standard auditory stimuli used in the experiments consisted of five clicks arranged at regular, 30.3 ms time intervals (corresponding to 33 Hz, thus we named the click-trains gamma quintets), while deviant stimuli (targets in the engaged condition) differed in that the third click was shifted towards the fourth (shift *range* = 15–30.3 ms, Figure 1). The 33 Hz repetition rate of the five clicks corresponds to the gamma frequency range of the EEG. This stimulus arrangement resulted in a hierarchically organized rhythmic stimulus structure on two coupled time scales [i.e., delta (1.6 Hz) and gamma (33 Hz), that was designed to examine whether the entrainment of ongoing neuronal oscillations can occur simultaneously in multiple frequency bands, a mechanism that was proposed as one of the cornerstones of speech perception and analysis (Schroeder et al., 2008; Ghitza, 2011; Giraud and Poeppel, 2012)].

### The Effect of Engagement on Responses to Click-Trains

To assess the general effect of engagement in the task on auditory responses, we statistically compared MUA response amplitudes to the gamma quintets in engaged vs. passive conditions within each experiment. Since we presented stimuli at two different loudness levels in both conditions (40 and 50 dB), we determined the effect of engagement separately for these. Across all recording sites, response onset latency to 50 dB attended click-trains was on average 7.41 ms (SD = 0.92 ms) and did not differ significantly between the active and passive conditions (Wilcoxon signed rank, *p* = 0.239). Since previous studies have shown that response onset varies across differently tuned regions in A1 (Mendelson et al., 1997; Kaur et al., 2004; Lakatos et al., 2005a; O’Connell et al., 2011), we tested this by comparing response onsets in A1 regions with best frequencies (BF) of 8 kHz or lower to response onsets in neuronal ensembles tuned to higher frequencies (*BF* >8 kHz). As predicted, response onset latencies in A1 sites tuned to lower frequencies were significantly longer than those in sites tuned to higher frequencies (17 vs. 31 recording sites, Wilcoxon rank sum, *p* = 0.0409). Interestingly, when we tested whether response onset latency was significantly different across left and right hemispheres (23 vs. 25 recording sites), we found that left hemisphere latencies were significantly shorter, albeit only on average by 0.74 ms (Wilcoxon rank sum, *p* = 0.0227).

Since, as described above, there was no significant difference in response onsets across different behavioral conditions, we measured MUA response amplitude averaged across all cortical layers in the timeframe from earliest response onset (6 ms) until 40 ms post-stimulus (160 ms). When we statistically compared response amplitudes within all experiments in the engaged vs. passive conditions (Wilcoxon rank sum with Bonferroni correction), we found that for both 40 and 50 dB click-trains, engagement resulted in significant response suppression in most A1 sites [*n* = 26 (54%) in the case of 40 dB and *n* = 32 (66%) in the case of 50 dB click-trains]. The upper traces in Figure 2A (upper panel) show the averaged responses of sites that showed significant engagement related response suppression to the stimulus trains presented at either loudness (*n* = 32, “suppression group”). The rest of the sites either showed no engagement-related response modulation at any loudness (*n* = 12), or a significant response enhancement (*n* = 3 in the case of 40 dB and *n* = 2 in the case of 50 dB trains). Since enhancement only occurred in a fraction of our experiments (0.08%), we pooled these sites with the “no response amplitude change” ones and termed them “no suppression group” (*n* = 16), the averaged responses of which are shown in the lower panel of Figure 2A. By observing the averaged responses of the suppression group (Figure 2A, upper), we noted that the effect of loudness only appears to affect the transient parts of the response, thus we performed quantitative analyses to verify this notion. Indeed, while blue brackets in Figure 2B denote significant differences between 40 and 50 dB fourth click related transient response amplitudes in the 8–14 ms post-click timeframe within the same attentional condition (i.e., passive or engaged), there is no significant difference between 40 and 50 dB related pre-click (−10 to −5 ms) amplitudes (Figure 2C). However, the effect of engagement is observable across the whole “response timeframe”: red brackets in Figure 2B denote significant differences between passive and engaged fourth click related transient responses at both stimulus intensities, and red brackets in Figure 2C denote significantly different pre-click (or inter-click) MUA amplitudes in passive vs. engaged conditions. We also noted that in these averaged responses, the effect of engagement on the transient responses to clicks corresponds to a 10 dB decrease in loudness (there was no significant difference between responses to 40 dB clicks in the passive and responses to 50 dB clicks in the engaged condition, *p* > 0.05, Kruskal-Wallis test with Tukey’s test).

To determine whether there is a relationship between the tuning of the neuronal ensembles and effect of engagement on click-train related responses, we sorted the recording sites according to their BF, which is displayed in Figure 2D. It is apparent that the “no suppression” group of sites mostly had BFs of 11 or 16 kHz, and never BFs lower than 5.6 kHz. As opposed to this, neuronal ensembles with engagement related suppression occurred in regions tuned to both high and low frequencies along the tonotopic axis. These two groups of sites were relatively evenly distributed across both hemispheres (Jarque-Bera test, *p* = 0.071, 10 vs. 6 non-suppressive sites in left vs. right hemispheres).

In an attempt to categorize the responses based on the effect of engagement, we examined the responses in laminar CSD profiles and at first, did not notice any apparent pattern. In fact, we were puzzled by the variability in engagement related effects. Figure 3 illustrates this by showing the CSD response profiles of three differently tuned A1 sites: one from the non-suppressive and two from the suppressive group. The CSD profiles of the non-suppressive site (*BF* = 16 kHz) are highly similar in the passive vs. engaged conditions (Figure 3A), with slightly higher averaged CSD response amplitudes in the supragranular layers, as illustrated by the traces to the right that show the CSD of selected electrode contacts from different cortical layers. Additionally, the baseline appears less “flat” (more tilted in the CSD traces) in the engaged condition in the supragranular layers. The MUA response of this particular site is enhanced in the engaged condition across all cortical layers. The next site (Figure 3B) is tuned to low frequencies (*BF* = 0.5 kHz), and as the laminar MUA profiles show, the MUA response to click-trains is suppressed across all layers in the engaged condition. Compared to the first site, the laminar CSD response in the passive condition appears overall larger in amplitude, with maybe a slight polarity difference in the supragranular layers. Similar to the first site, the baseline appears more tilted on the selected supragranular channel in the engaged condition (CSD traces), although in the opposite direction. The third site shown in (Figure 3C) was tuned to 8 kHz, and since this site also belongs to the suppressive group, the MUA response appears attenuated across all layers in the engaged condition. The most apparent difference between the laminar CSD response profiles in the two conditions is that in the supragranular layers, the source over sink pattern in the passive condition appears flipped to sink over source in the engaged condition in this third site.

### The Pattern of Delta and Gamma Frequency Entrainment Across A1

To quantify the observed CSD differences between the two conditions, we measured the mean phase and phase consistency (inter-trial phase coherence or ITC) of supragranular neuronal activity at the delta and gamma frequencies that corresponded to the repetition rates across and within click-trains (1.6 and 33 Hz respectively). Our reasoning was that several previous studies have shown that modulating the phase and/or strength of oscillatory entrainment can modulate responses to attended tones (Lakatos et al., 2008, 2013; O’Connell et al., 2014). Thus, assuming that the phases measured reflect the phase of entrained oscillatory activity as opposed to evoked type, *de novo* generated neuronal activity, the pattern of phase alignments could reveal a potential mechanism of response suppression in the engaged condition. To verify this assumption, we compared the amplitudes of delta and gamma band neuronal activity in data that were recorded in the absence of stimulation (spontaneous activity) to delta and gamma amplitudes measured in the passive and engaged conditions.

**Figure 4:**
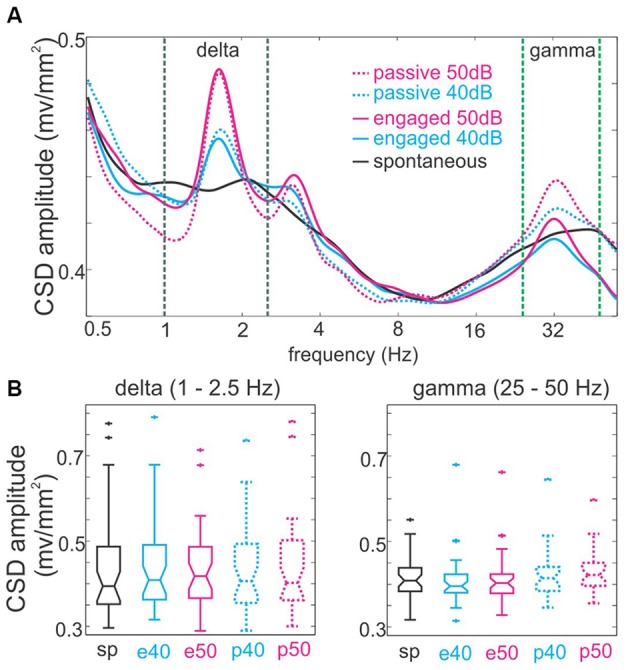
**The amplitude of spontaneous and auditory stimulus stream related delta and gamma band neuronal activity. (A)** The spectra averaged across all experiments (*n* = 48) show that the presentation of click-train streams results in discrete peaks that correspond to the repetition of clicks within click-trains (33 Hz) and the rate at which click-trains occurred in the stream (1.6 Hz). These peaks are larger in the case of louder streams. The dotted lines mark the delta (black, 1–2.5 Hz) and gamma (green, 25–50 Hz) frequency bands. **(B)** Boxplots show pooled amplitudes of neuronal activity averaged in the delta (left) and gamma (right) frequency bands.

**Figure 5:**
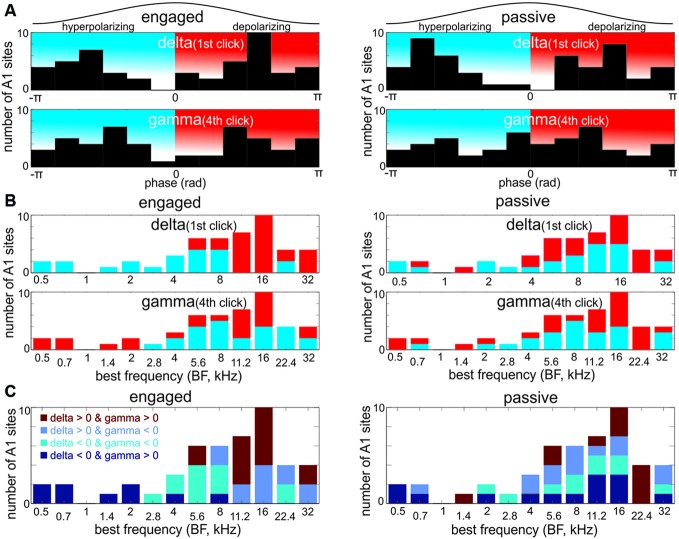
**The distribution of pre-stimulus delta and gamma oscillatory phases across differently tuned A1 regions. (A)** Histograms show the distribution of delta phases measured at the onset of the first click (0 ms) and gamma phases measured at the onset of the fourth click (90.9 ms) in the engaged and passive conditions. Phases were measured at the delta and gamma frequencies that correspond to the stimulus onset asynchrony (SOA) between and repetition rate within click-trains (1.6 and 33 Hz respectively). Black traces above the histograms represent one oscillatory cycle. Therefore, the histograms illustrate where in the delta/gamma cycles the first/fourth clicks tend to occur. It is apparent that both delta and gamma phases in the engaged, and delta phases in the passive condition have a bimodal distribution, indicating that click related inputs tend to fall on either of two preferential phases either the upward deflection (hyperpolarizing phase) or downward deflection (depolarizing phase). **(B)** The BF distribution of sites with hyperpolarizing and depolarizing pre-stimulus phases. Note that sites with depolarizing pre-stimulus delta phases in the engaged condition have high BFs. Also note that the gamma phases in high frequency tuned sites are mixed, while low frequency sites have depolarizing gamma phases at the onset of the fourth click. Importantly, the BF distribution of sites with depolarizing and hyperpolarizing delta and gamma phases is markedly different only in the engaged condition. **(C)** Theoretically, delta and gamma phases can combine in four different ways, and as the histograms show, they indeed do. Note that the distribution of sites with depolarizing delta and gamma phases (*delta* > 0 and *gamma* > 0) in the engaged condition appears to be very similar to the BF distribution of sites with no significant engagement related suppression (Figure 2D).

Figure 4A shows the spectrograms of supragranular neuronal activity (CSD) in the absence of stimulation and in different task conditions during the presentation of click-trains. While it is obvious that at both delta and gamma stimulation rates, the amplitude spectrum of neuronal activity is “peaked” compared to the spontaneous spectrum, note that this is paired with lower amplitudes around the peak in the auditory stimulus stream related spectra resulting in no significant net amplitude change in the delta and gamma bands (Kruskal-Wallis test, *p* = 0.9519 and *p* = 0.1549 respectively, Figure 4B). Rather, the peaks most likely represent a reorganization of oscillatory activity to match relevant temporal scales that results in a concentration of energy at the frequencies that correspond to the repetition rates of stimuli. In other words, the peaks mostly signal neuronal activity that is less variable in frequency in the delta and gamma bands, which has been shown to be characteristic of oscillatory entrainment (Lakatos et al., 2013; Zoefel and Heil, 2013). Nevertheless, we cannot exclude the possibility that evoked type activity contributes to the measured spectra. As a matter of fact it is likely, especially in the case of 50 dB click-trains: the harmonic at double the stimulation rate (~3.2 Hz) can be a strong indication of evoked type activity that “distorts” the sinusoidal waveform that is characteristic of entrainment. Previous studies indicate that the amplitude ratio of evoked type (added) neuronal activity to the ongoing neuronal activity determines the “distorting” effect of evoked responses on phase measurements of the ongoing neuronal oscillations (Lakatos et al., 2013). Since based on the spectra, this ratio is very small in the case of 40 dB click-trains (on average 1.00 in the delta and 0.99 in the gamma range for 40 dB, and 1.03 in both the delta and gamma frequency ranges for 50 dB, with the gamma ratio difference significant (Wilcoxon signed rank test, *p* < 0.001)), we only analyzed delta and gamma phases related to the lower intensity stimuli in engaged vs. passive conditions. To further minimize confounding effects of the response evoked by the onset of the click-train, as in previous studies (Lakatos et al., 2013; O’Connell et al., 2014), we applied linear interpolation to the data in the 5–150 ms timeframe before we measured delta phases (see “Materials and Methods” Section). Furthermore, while we measured delta phase at stimulus onset (0 ms), gamma phases were measured at the time of the fourth click (90.9 ms in non-interpolated data) to get a more reliable estimate of the entrained gamma phase. Figure 5A displays the histograms of mean delta (top) and gamma (bottom) phases across all experiments in the engaged (left) and passive (right) task conditions. It is apparent that the phase distributions are bimodal in most cases (except gamma phases in the passive task condition). One group of phases peaks between –pi and 0, on the upward deflection of the neuronal oscillation, while the other group is centered on the downward deflection. Our previous studies analyzing supragranular CSD oscillations in the same laminar position have provided evidence that while the upward deflection corresponds to the low excitability, or hyperpolarizing phase of cortical neuronal oscillations, the downward deflection corresponds to the high excitability, depolarizing phase (e.g., Lakatos et al., 2005b). This was determined indirectly by analyzing fluctuations in the level of spontaneous (incidental) neuronal ensemble firing and gamma oscillatory amplitudes, both of which are highest on the depolarizing phase of ongoing oscillations. To verify this in our current data, we first grouped MUA and gamma oscillatory amplitude (measured in the 25–50 Hz band) averaged across all layers into two bins based on the phase of delta oscillatory activity: MUA and gamma amplitude during delta phases from –pi to 0 (upward deflection) fell into one bin, while the rest (during delta phases from 0 to –pi, the downward deflection) were put into the second bin. We found that even though the difference between bins was on average very small (0.8% difference for MUA and 2.9% for gamma), both MUA and gamma frequency laminar activity was significantly larger during the downward slope of the supragranular delta oscillation (Wilcoxon signed rank, both *p* < 0.0001), confirming that this is indeed the high excitability or depolarizing phase of delta (similar to Figure 4A of Lakatos et al., 2005b). Similarly, although not as highly significant, we found that MUA was significantly higher in amplitude during the depolarizing phase of gamma band oscillatory activity (Wilcoxon signed rank, *p* = 0.015). This is physiologically plausible, because while negative trending values in the CSD represent net inward transmembrane current which signals a depolarization of the local neuronal ensemble (hence the name depolarizing phase), positive trending values signal net outward current and thus hyperpolarization (hyperpolarizing phase). Therefore, we decided to use these two phase bins to tag delta and gamma phases (Figures 5A,B).

To examine the relationship between the frequency tuning of A1 neuronal ensembles and the phase of delta and gamma entrainment, we created bar graphs with the phase of oscillations color coded (Figure 5B, red = depolarizing, blue = hyperpolarizing phase). We found that in the engaged condition, attended click-trains in sites with higher BFs entrained delta oscillations to their depolarizing phase, while in sites tuned to lower frequencies delta oscillations were entrained to their hyperpolarizing, low excitability phase. The BF distribution of the sites entraining to the click trains with a depolarizing delta phase was significantly different from that of the sites entraining with a hyperpolarizing delta phase (Wilcoxon rank sum test, *p* < 0.001, depolarizing delta phase median *BF* = 16 kHz, hyperpolarizing delta phase median *BF* = 4 kHz). The mean phase of gamma oscillatory activity during the engaged condition showed a more complicated pattern: in sites tuned to ≤2 kHz and to 11–16 kHz we measured depolarizing phases, while hyperpolarizing gamma phases appeared biased towards sites tuned to frequencies surrounding 11–16 kHz, however there was no significant difference in the BF distribution of sites exhibiting depolarizing or hyperpolarizing pre-stimulus gamma phases (Wilcoxon rank sum test, *p* = 0.2). In the passive condition, for both delta and gamma pre-stimulus phases, there was no obvious BF distribution pattern (Wilcoxon rank sum test, both *p* values > 0.2).

Next, we examined how the phases of delta and gamma entrainment “combine” in each site. One possibility is that, for example, the depolarizing phase of delta always co-occurs with the depolarizing phase of gamma in one group of sites, and the hyperpolarizing phases of the entrained oscillations combine in another group of sites. However, two other combinations are theoretically possible: hyperpolarizing gamma phases combined with depolarizing delta, and vice versa. In fact, when we looked at the combination of delta and gamma phases, we found that all four possible combinations occur. Furthermore, these seem to be grouped in sites tuned to similar frequencies in the engaged condition (Figure 5C): e.g., while hyperpolarizing delta and depolarizing gamma phases co-occur in regions tuned to 2 kHz and below, depolarizing delta and gamma phases co-occur overwhelmingly in regions tuned to 11–16 kHz (11 out of 17 sites, or 64.7%). We noted that the BF distribution of sites in this latter group shows remarkable similarity to the BF distribution of sites in the no-suppression group (Figure 2D). When we compared the “depolarizing delta-gamma” (*n* = 15) and no-suppression group of sites (*n* = 16), we found that 12 of the sites were indeed the same.

**Figure 6:**
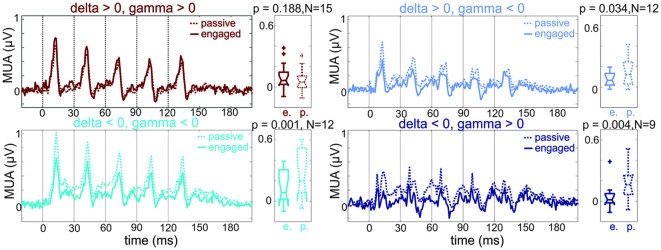
**MUA responses in A1 regions with differing pre-stimulus phases.** Pooled MUA responses of sites with different pre-stimulus delta-gamma phase combinations (same groups of sites and same color coding as sites in the engaged condition of Figure 5C). Boxplots to the right show the MUA response amplitudes across sites averaged in the 60–160 ms time interval. While the first group of sites shows no engagement related suppression, the other three groups of sites do. Note that the patterns of suppression seem to match the delta-gamma phase combinations remarkably well: while the suppression is maximal at the times of the individual click related responses in the depolarizing delta—hyperpolarizing gamma group (upper right panel, light blue), the suppression is more sustained in the groups where pre-stimulus delta is in a hyperpolarizing phase (lower panels, cyan and dark blue). Wilcoxon signed rank test, *p* < 0.01, N = number of A1 sites in each group.

This indicates that the majority of sites in the depolarizing group show either no response amplitude change or a response enhancement in the engaged vs. passive condition. It also follows that sites in the other three phase combination groups belong to the group of sites with significant engagement related MUA response suppression. To verify this and to uncover any multiscale entrainment specific differences, we pooled MUA responses based on delta-gamma phase combination into four groups, and compared response amplitudes in the later portion (60–160 ms) of the response in the engaged vs. passive conditions (Figure 6). We found that as predicted, with the exception of the depolarizing delta-gamma group, engagement resulted in significant response suppression (Wilcoxon signed rank test: depolarizing delta-gamma group: *p* = 0.118, all other groups: *p* < 0.05).

While in some cases, delta and gamma phase combinations differ across engaged and passive conditions (Figure 5C), mean delta and gamma phase in the majority of sites does not change (e.g., sites tuned to 0.5 kHz). Thus we asked whether phase consistency across single trials was different in engaged vs. passive conditions, since it has been shown that engagement results in a stronger phase reset and entrainment of ongoing neuronal activity (Lakatos et al., 2009, 2013; O’Connell et al., 2014), and a stronger enforcement of suppressive phase patterns via entrainment could in theory result in significant suppression of responses. We found that as expected, both delta (1.6 Hz) ITC measured at stimulus onset, and gamma (33 Hz) ITC measured at the time of the fourth click in the click-train was significantly greater in the engaged condition (Wilcoxon signed rank, both *p* < 0.001). While delta and gamma phase consistency was significant in all sites in the engaged condition, in the passive condition, delta and gamma ITC was not always significant (Rayleigh test, Bonferroni corrected *p* < 0.0005 in 21 sites for delta and 25 sites for gamma). This most likely indicates that the passive condition is a mixed ignore/attend condition, since we did not employ a selective attention task where the animals had to ignore the click trains in order to attend to an alternate stimulus stream. The fact that in most cases in the passive condition, delta and gamma phases were not significantly biased also explains why mean phase distributions across differently tuned sites appear less congregated around sites with similar BFs (Figures 5B,C), since the mean of non-significant phase distributions can be considered random. Thus it is not phase distribution *per se* that is different between passive and engaged conditions but the strength of entrainment.

### Engagement Related Hemispheric Asymmetry

**Figure 7:**
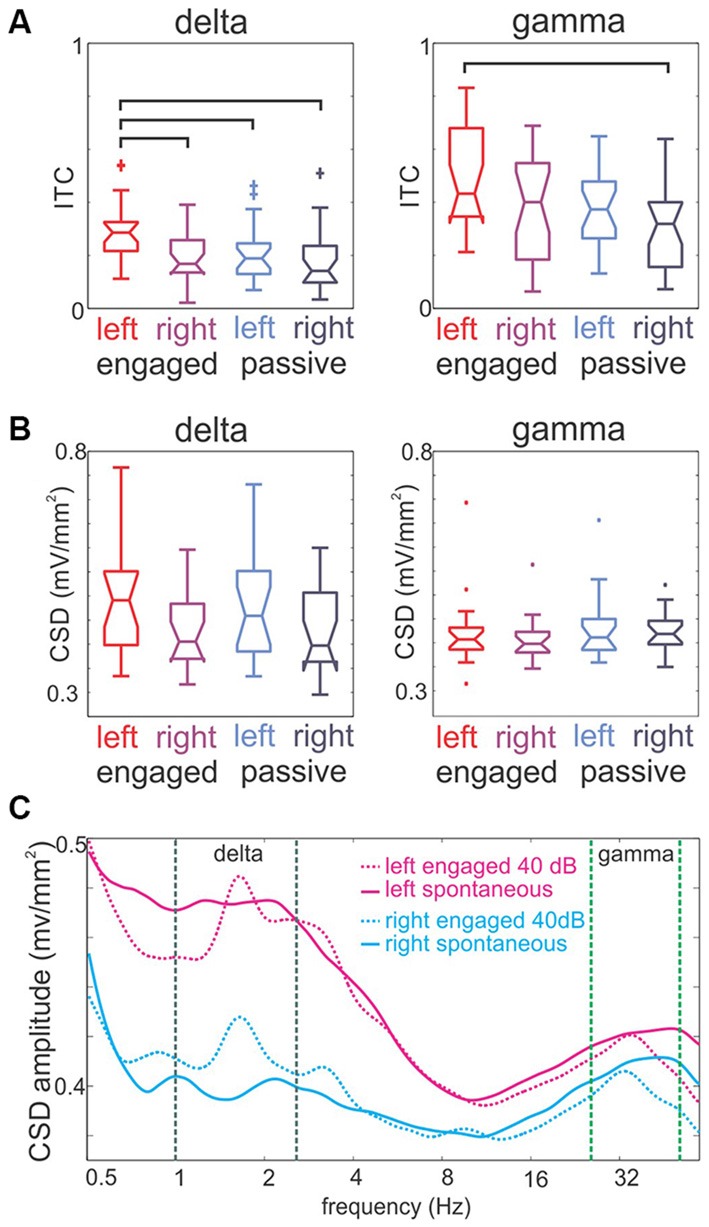
**Hemispheric lateralization of intertrial phase coherence and the amplitude of delta and gamma oscillations. (A)** Pooled delta and gamma (1.6 and 33 Hz) Inter-trial coherence (ITC) values in left and right A1 sites in engaged and passive conditions. Generally, engagement results in a significant ITC increase (see “Results” Section), and both delta and gamma ITC is higher in left hemisphere sites. Brackets mark significant differences (Tukey’s test, *p* < 0.01). **(B)** Pooled delta and gamma oscillatory amplitudes in left and right hemisphere A1 recordings in engaged and passive conditions. Both delta and gamma amplitude values follow the same trend as ITC, but none of the effects are significant. **(C)** Spectra of ongoing and 40 dB click-train stream related activity in the engaged condition.

Recent human research suggests that the processing of auditory stimuli structured at different timescales is hemispherically asymmetric (for a review, see Giraud and Poeppel, 2012). We designed our stimuli in part to mimic the multi-temporal scale organization aspect of vocalizations, and thus, we were interested in the question of whether there is evidence of hemispheric asymmetry in the entrainment of fast and slow oscillations. To determine this, we pooled our delta and gamma ITC measures according to task condition and hemisphere. As Figure 7A shows, we found that delta ITC related to click-train streams in the engaged condition was significantly greater in left hemisphere sites than delta ITC in either left or right hemisphere sites in the passive condition (Tukey’s test, *p* < 0.01). Importantly, left delta ITC in the engaged condition was also greater than right delta ITC in the same condition. These data indicate that there is a hemispheric asymmetry in the strength of delta entrainment due to engagement. A similar trend is apparent for gamma ITC, however left hemisphere gamma ITC in the engaged condition was only significantly larger than right hemisphere gamma ITC in the passive condition (Tukey’s test, *p* < 0.01). When we compared oscillatory amplitudes across hemispheres and task conditions, at the delta and gamma frequencies that corresponded to the SOA (1.6 Hz) and the repetition rate of click in the gamma quintets (33 Hz), there was a trend towards a similar leftward bias but no significant effects (Kruskal-Wallis test, *p* > 0.05; Figure 7B). Taken together these data indicate that left A1 exhibits greater stimulus structure related delta and gamma band oscillatory activity, and that engagement in the task enhances hemispheric differences in the oscillatory neuronal activity of the supragranular layers.

Importantly, as our results above foreshadowed (Figure 4), larger delta and gamma amplitudes related to click-trains in the left A1 are not due to larger evoked responses. As the spontaneous and stimulus-related spectra in Figure 7C show, the difference between spontaneous and auditory stimulus-related delta amplitudes at the rate of stimulation is actually larger in the right hemisphere indicating that perhaps in the right hemisphere evoked activity contributes more substantially to the delta peak in the spectrum of stimulus-related activity. Contrary to this, in the left hemisphere there is no net amplitude change between the two conditions, indicating that most likely entrainment is responsible for the delta peak at the stimulation rate.

## Discussion

Our main hypothesis was that when the broadband frequency spectrum of attended stimuli is irrelevant for an auditory task, ongoing oscillatory activity across all of A1 would be entrained by the auditory stimuli so that its high excitability, depolarizing phase would be aligned to the stimuli’s onset, maximally amplifying auditory responses. However, to our surprise we found that even though all of A1 entrained its ongoing neuronal oscillations to the temporal structure of attended stimuli on two timescales, delta and gamma, the net effect of entrainment on auditory responses was mostly suppressive. Both the pattern of entrainment and engagement related response amplitude modulation differentiated sites across the tonotopic map in A1: ongoing oscillations of most neuronal ensembles within the 11–16 kHz region of A1 were entrained by the stimuli to their high excitability phases when monkeys engaged in the auditory task, and responses to task relevant stimuli in these sites were either enhanced or not significantly modulated compared to responses in the passive condition. In contrast, in most neuronal ensembles outside of this A1 region, either delta (for sites further from the 11–16 kHz region) or gamma (for sites closer to the 11–16 kHz region) oscillations were entrained to their low excitability phases in the engaged condition (see Figure 5B), which co-occurred with significant response suppression compared to the passive condition. Congruent with a more organized pattern of entrained delta and gamma phases, stimulus timing related delta and gamma phase consistency (ITC) were both significantly larger in the engaged compared to the passive condition. Taken together, our findings indicate that neuronal ensembles tuned to the higher frequency portion of the audible spectrum play a central role in the sensory representation and processing of relevant broadband transient sounds, like auditory clicks. Additionally we found that engagement-related oscillatory entrainment on both slow and fast time scales was stronger in left hemisphere A1 sites, albeit only delta frequency effects were significant.

### Mechanisms of Predictive Response Suppression

**Figure 8:**
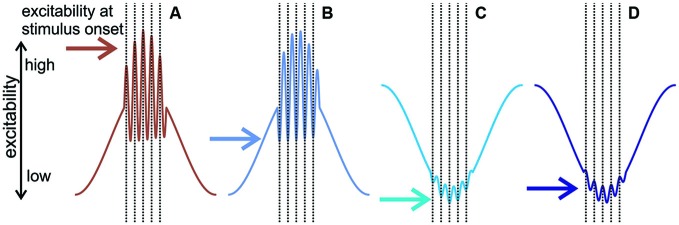
**The predicted effect of delta—gamma phase amplitude coupling and multiscale entrainment to various phases on neuronal excitability. (A)** Diagram of neuronal excitability when delta and gamma are both entrained to their depolarizing phases: depolarizing delta results in large amplitude gamma, and as gamma is also at its depolarizing phase at the time of the stimuli, this results in a high excitability state when stimuli are expected to occur. **(B)** Depolarizing delta/hyperpolarizing gamma: the amplitude of gamma is still large but as it is entrained to its hyperpolarizing phase, this results in a relatively low neuronal excitability state at the onset of stimuli, which should lead to transient MUA response suppression. In **(C,D)** delta is entrained to its hyperpolarizing phase and therefore gamma amplitudes are small, thus gamma phase (hyperpolarizing in **(C)** and depolarizing in **(D)** does not play a major role in neuronal excitability modulation). Therefore, we predict sustained MUA suppression due to the hyperpolarizing phase of delta in both scenarios.

Our result of oscillatory entrainment across multiple frequency bands is in line with a previous study (Henry et al., 2014), but how does the multiscale entrainment of delta and gamma band oscillations result in a net suppressive effect in most neuronal ensembles? Previous studies provide a wealth of evidence, which we verified in the present study, that both delta and gamma oscillations have depolarizing (or high-excitability) and hyperpolarizing (or low excitability) phases (for a review, see Young and Eggermont, 2009). Our results show that in about half of the A1 sites examined, delta and gamma band oscillations were entrained by the clicks to the former, while the other half to the latter phase (Figure 5A). On a first hunch, this should result in an equal distribution of response enhancement and suppression across sites, which is not what we found (out of the 48 sites we recorded from, 16 sites showed no suppression while 32 sites exhibited suppression). The reason for this is twofold: first, hyperpolarizing and depolarizing phases of entrained delta and gamma oscillations are not always paired; we found that they co-occur in all four possible combinations. Second, delta and gamma oscillations are phase amplitude coupled, meaning that the phase (i.e., high/low excitability) of a lower frequency oscillation determines the amplitude (large/small) of a higher frequency band oscillation (Buzsáki et al., 2003; Lakatos et al., 2005b, 2008; Canolty et al., 2006), as shown by the significantly smaller gamma amplitude on the hyperpolarizing phases of delta oscillations in our data. Now let us consider the four phase combinations taking into account phase amplitude coupling. Diagrams in Figure 8 show the predicted excitability of neuronal ensembles entraining with the different delta-gamma phase combinations at click onset. If the depolarizing phases of delta and gamma co-occur, since delta is in a high excitability phase, the amplitude of gamma oscillations will be large, and as they are also being entrained to their high excitability phase by the clicks, this should result in a high excitability state of the neuronal ensemble when stimuli are predicted to occur, and thus enhanced response amplitudes (e.g., Figure 8A). However, if gamma oscillations entrained to their hyperpolarizing phases ride on the depolarizing phase of delta, gamma amplitude will still be large, but the hyperpolarizing phase of gamma will negate the depolarizing effect of delta, resulting in a net hyperpolarized state of the local neuronal ensemble in short, precisely timed temporal windows of low excitability centered on the clicks (e.g., Figure 8B), which should result in transient response suppression (Figures 8B, 6, upper right panel). In the remaining two categories of sites, since delta is entrained to its hyperpolarizing phase by the click trains, and thus gamma amplitudes will be low, the phase of gamma does not play an effective role in modulating excitability (Figures 8C,D). Therefore, the net effect should be long time-scale predictive suppression related to the hyperpolarizing phase of delta. In support of this, a visual inspection of the MUA responses in Figure 6 indicates that while mainly the transient part of the click-train related responses is suppressed in sites with depolarizing delta and hyperpolarizing gamma entrainment (Figure 6, upper right panel), suppressive effects appear much more tonic (with a longer time-constant) in hyperpolarizing delta sites (Figure 6, lower two panels).

### The Effect of Engagement on Neuronal Activity in A1

Contrasting task engaged and passive conditions, like in the present study, is often used in animal studies to investigate behavioral state related changes in neuronal activity. Regardless of sensory modality, a common finding in rodent studies when comparing responses to stimuli in engaged vs. passive states is that responses are suppressed in the active behavioral condition (Fanselow and Nicolelis, 1999; Castro-Alamancos, 2004; Crochet and Petersen, 2006; Ferezou et al., 2006; Otazu et al., 2009). This is usually interpreted as a sharpening or refinement of the sensory input. Our main finding is in line with these previous studies in that the overall effect of engagement in the task is response suppression. While not tested quantitatively, our data suggest that, at least when stimuli are broadband, engagement related MUA suppression is biased towards lower frequency tuned A1 neuronal ensembles (Figure 2D). This differs from the results of a previous study in rat auditory cortex, which found no tonotopic organization of engagement related suppression (Otazu et al., 2009). Our data also reveal a candidate mechanism for the engagement related sharpening of the sensory representation: the modulation of subthreshold neuronal ensemble activity via the alignment of rhythmic excitability fluctuations to the temporal structure of relevant auditory stimuli. This alignment, the entrainment of neuronal oscillations occurs in the passive condition as well (similar to Lakatos et al., 2005b), but to a significantly lesser degree, and in a less organized pattern.

### The Importance of Broadband Transient Sounds in Auditory Processing

In one of our earlier studies, we proposed that the brain “models” the spectrotemporal properties of selectively attended auditory stimuli and stimulus streams in the form of temporally evolving phase patterns arranged in space across topographically organized A1 neuronal ensembles (Lakatos et al., 2013). This in turn forms the basis for enhancing and stabilizing the representation of attended auditory information at the expense of irrelevant, background auditory stimuli. The present study, however, found that the physical frequency spectrum of the auditory click is represented in a “distorted” form, since its representation is mostly enhanced in high, while suppressed in low frequency regions of A1. Thus we speculate that it is possible that sharp transients, like clicks or formant transitions, represent a special category of auditory stimuli for which preserving an accurate frequency representation is less important. Rather, the main role of these “acoustic edges” could be to orchestrate the coherent multiscale entrainment of neuronal oscillations across differently tuned A1 neuronal ensembles, thereby setting up a spatiotemporal excitability pattern that is ideal for the parsing and processing of relevant auditory content mainly contained in the lower frequency spectrum e.g., speech (Fletcher, 1948; Peelle et al., 2013). In this theoretical framework, acoustic edges would form the temporal context that enables the most efficient processing of the acoustic content by modulating ongoing neuronal oscillations.

Broadband transient sounds are common features of speech in humans (e.g., stop consonants) and conspecific vocalizations in monkeys (May et al., 1989; Wang et al., 1995). Aside from communication sounds, they also occur frequently in the acoustic environment, in which case they mostly indicate something alerting requiring quick action (e.g., the snap of a twig). Thus, in theory, it would be advantages to process these sounds via a fast dedicated auditory processing hierarchy of neuronal ensembles. Indeed, there are neurons in the posteroventral cochlear nucleus that are specialized in responding to broadband transients, called octopus cells. The main function of the octopus cells appears to be the integration of the cochlear activation via the summation of orderly dendritic synaptic activation, which compensates for the traveling wave delay of the cochlea (Rhode et al., 1983; Golding et al., 1999; Oertel et al., 2000; McGinley et al., 2012). These cells fire extremely fast and are very precise temporally (Rhode and Smith, 1986). Their output is transmitted via a separate ascending pathway mainly to the contralateral ventral nucleus of the lateral lemniscus, a pathway which appears to be much more prominent in humans (Adams, 1997). Interestingly, it has also been shown that octopus cells integrate cochlear inputs over about 1/3 of the audible spectrum (Oertel et al., 1990; Golding et al., 1995, 1999), which does correspond to the BF “spread” of the no-suppression group in our data. Thus, we hypothesize that the group of non-suppressive sites in A1 that are tuned to 11–16 kHz might form the first cortical stage of the ascending auditory pathway specialized in rapidly processing broadband transient sounds.

Besides rapid alerting, this “transient specialized system” together with the modulation of ongoing neuronal activity across A1 could play a crucial role in the processing of complex acoustic patterns like communication sounds. As suggested earlier, one role could be parsing (Ghitza and Greenberg, 2009; Buzsáki, 2010; Ghitza, 2011; Giraud and Poeppel, 2012): entrained delta/theta phase related suppression of the neuronal ensembles processing lower frequency speech sounds (<5 kHz) would be an efficient mechanism to segment continuous speech, while gamma, which is “nested” in delta/theta assists in the modulation of excitability and thus stimulus processing on shorter timescales (e.g., phonemic scale).

Expanding on the “gamma nested in delta/theta” model (Ghitza, 2011; Giraud and Poeppel, 2012), we propose that complementary to parsing, the other main role of transients in speech might be to prepare lower frequency cortical areas for the processing of band-limited sounds. This would be important since speech has regularly and predictably interchanging broadband transients (i.e., consonants) and more “tonal” elements whose main spectral energy is band limited and is usually below 5 kHz (i.e., vowels). Our results provide evidence that this could be achieved by resetting and entraining ongoing oscillatory activity to their low excitability phases in most A1 areas (i.e., outside the 11–16 kHz region): as a consequence, the high excitability, depolarizing phase of ongoing oscillations will be centered on acoustic elements positioned between sharp transients (acoustic edges). In fact, this notion is in line with the findings of a recent behavioral study where the ability of subjects to detect a 1 kHz tone modulated in a fashion that was antiphasic to the amplitude-modulated broadband noise stream that preceded it (Hickok et al., 2015). This “antiphasic oscillation of perceptibility” is most likely due to the fact that the broadband noise (like click) entrained oscillations in high frequency regions of auditory cortex to high-, but low frequency regions to low-excitability phases. Thus the detection of a low frequency tone would be enhanced at the trough, not the peak of the amplitude modulated noise. Based on our previous studies (Lakatos et al., 2013; O’Connell et al., 2014), lower frequency speech elements (vowels) could also reset delta and gamma oscillations to their depolarizing phases in low frequency regions and to their opposite, hyperpolarizing phases in high frequency regions. This in turn would prepare these areas for an upcoming high frequency element or sharp transient. Therefore, we hypothesize that within a syllable, which occurs at a delta/theta rate (Greenberg et al., 2003), and is usually constructed of a consonant (high frequency element) and a vowel (low frequency element; Poeppel, 2003), counterphase entrained oscillations in the delta/theta band across all of A1 are reset twice, once by high frequency (consonant related) and once by low frequency (vowel related) inputs. This would provide a highly adaptive very precise dual timing mechanism for the synchronization of neuronal oscillations to attended speech that is thought to be a key element of speech processing and perception (see below), which should be especially helpful in noisy environments, like at a cocktail party. An everyday observation in support of the above hypothesis is that it is close to impossible to make out someone’s speech over the phone (which transmits acoustic signals only below 5 kHz) when background noise is high or when multiple people are speaking. We speculate that the presence of noise results in deterioration in performance not only due to missing acoustics masked by the noise (see Appendix in Ghitza, 2011; also Shamir et al., 2009), but also as a result of the missing half of the temporal context contained in the high frequencies of the auditory spectrum, which prevents the precise alignment of nested oscillations to speech. A similar mechanism could explain aging related deficits in speech comprehension, which manifests stronger in the presence of environmental noise, since ageing often results in high frequency hearing loss (reviewed by Pichora-Fuller and Souza, 2003).

### Evidence for Hemispheric Functional Lateralization in Non-Human Primates

Both delta and gamma oscillations, along with theta have been proposed to be important in the processing of speech and species specific communication (Schroeder et al., 2008; Ghitza, 2011; Giraud and Poeppel, 2012). We found that multiscale oscillatory entrainment at these rates shows greater phase consistency at the time attended auditory stimuli occur in left A1, indicating a stronger involvement of left hemisphere oscillatory activity. This finding provides support for the functional asymmetry of left and right auditory systems at the level of their first cortical processing stage, primary auditory cortex, and possibly indicates that the precursor of left hemisphere association with speech is present in non-human primates. Previous studies in monkeys provide behavioral (Petersen et al., 1978; Ghazanfar et al., 2001), ablation (Heffner and Heffner, 1984) and neuroimaging (Poremba et al., 2004) evidence for hemispheric lateralization for the processing of species specific communication. Anatomical studies in new and old world monkeys also found evidence for a leftward asymmetry (Heilbroner and Holloway, 1988; Gannon et al., 1998, 2008). Nevertheless, since the results relating to functional asymmetry are scarce, the hypothesized functional lateralization of auditory processing is still unresolved in non-human primates. To our knowledge, our study is the first to provide electrophysiological evidence for such hemispheric lateralization in monkeys. Importantly, our left and right measures are directly comparable since most data was recorded simultaneously in left and right primary auditory cortices.

We found that the hemispheric asymmetry in the strength of delta and gamma entrainment only became significant in the engaged condition. Additionally, we found no indication of an asymmetry in the strength of delta entrainment in a previous study (O’Connell et al., 2014), where monkeys were presented with a rhythmic stream of pure tones and were performing a frequency deviant detection task. Thus it appears that in macaques, functional asymmetry becomes apparent when spectrotemporally more complex stimuli are used and the subjects are engaged in a task where these stimuli are relevant. In fact, human studies that show indications for functional asymmetry using electrophysiological recordings and/or neuroimaging utilized similar spectrotemporally complex, rhythmic stimuli (Boemio et al., 2005; Jamison et al., 2006; Giraud et al., 2007; Obleser et al., 2008; Morillon et al., 2010), even though in some of these studies stimuli were presented in a passive condition. We speculate that in humans, hemispheric asymmetry might be structurally more solidified via evolution, which is why functional differences can be revealed even in a passive state. Another difference between human findings and our results in monkeys is that while in our data, entrainment on both long and short time-scales was left lateralized, most human studies find that slower (delta-theta) modulations of neuronal activity related to the temporal structure of the acoustic input are lateralized to the right hemisphere (e.g., Luo and Poeppel, 2012). We speculate that one reason for the difference might be stronger evoked type responses at the rate of stimulation in the right hemisphere, which would bias both neuroimaging and electrophysiological measurements. Although a thorough analysis of this proposition is beyond the scope of the present study, the spectrograms in Figure 7C do provide some support for this notion: while spontaneous low frequency oscillatory amplitude is smaller in right hemisphere recordings, the amplitude increase related to auditory stimuli in the delta band is larger. In the future, it will be important to conduct human experiments with near threshold auditory stimuli so that the effect of evoked type responses would be negligible. Nevertheless, despite some discrepancies, our results provide support that, similar to humans, relevant spectrotemporally complex rhythmic stimuli are processed asymmetrically by the left and right hemispheres. This result suggests that the functional-anatomical precursor to the machinery that enables speech perception and production might be present in non-human primates, at least at lower cortical stages.

## Conclusion

Our findings indicate that attended broadband stimuli organized on multiple timescales (i.e., repetitive click-trains) result in a multi-scale entrainment of ongoing oscillations across all of A1, and that the phases of entrainment of low and high frequency oscillations are independent of each other. Nonetheless, the intricate combination of low and high excitability phases in differently tuned neuronal ensembles results in a predominantly suppressive effect on auditory responses to click-trains, except in a subset of high frequency neuronal ensembles of A1. We hypothesize that the opposite sign excitability modulation of high vs. low frequency representation related to broadband transients could set the stage for the predictive processing of alternating high vs. low frequency elements of complex acoustic stimuli like speech. In this theoretical framework, oscillatory alignment to speech would be supported by a highly adaptive dual timing mechanism: both high (broadband) and low frequency elements would reset counterphase oscillations across all of A1 within the same delta/theta cycle at time points separated by only a half delta/theta cycle. Additionally, evidence of superior phase consistency of entrained oscillations in left A1 provides support for functional hemispheric asymmetry even at the earliest auditory cortical processing stage and remarkably even in non-human primates.

## Funding

This research project was funded by NIH grants RO1DC012947 and RO1DC011490.

## Conflict of Interest Statement

The authors declare that the research was conducted in the absence of any commercial or financial relationships that could be construed as a potential conflict of interest.
